# Telomere Lengthening and Other Functions of Telomerase 

**Published:** 2012

**Authors:** M.P. Rubtsova, D.P. Vasilkova, A.N. Malyavko, Yu.V. Naraikina, M.I. Zvereva, O.A. Dontsova

**Affiliations:** Lomonosov Moscow State University, Chemistry Department,; Belozersky Institute of Physicochemical Biology, Lomonosov Moscow State University; Lomonosov Moscow State University, Faculty of Bioengineering and Bioinformatics

**Keywords:** telomerase, reverse transcriptase, telomeres, mitochondria, DNA damage, gene expression

## Abstract

Telomerase is an enzyme that maintains the length of the telomere. The telomere length specifies the number of divisions a cell can undergo before it finally dies (i.e. the proliferative potential of cells). For example, telomerase is activated in embryonic cell lines and the telomere length is maintained at a constant level; therefore, these cells have an unlimited fission potential. Stem cells are characterized by a lower telomerase activity, which enables only partial compensation for the shortening of telomeres. Somatic cells are usually characterized by the absence of telomerase activity. Telomere shortening leads to the attainment of the Hayflick limit, the transition of cells to a state of senescence. The cells subsequently enter a state of crisis, accompanied by massive cell death. The surviving cells become cancer cells, which are capable both of dividing indefinitely and maintaining telomere length (usually with the aid of telomerase). Telomerase is a reverse transcriptase. It consists of two major components: telomerase RNA (TER) and reverse transcriptase (TERT). TER is a non-coding RNA, and it contains the region which serves as a template for telomere synthesis. An increasing number of articles focussing on the alternative functions of telomerase components have recently started appearing. The present review summarizes data on the structure, biogenesis, and functions of telomerase.

## INTRODUCTION 

The genetic information in eukaryotic cells is stored in linear DNA molecules known as chromosomes [1]. It was revealed as early as in the 1930s that the behavior of the whole chromosome and its fragments in cells varies. Torn chromosomes can fuse with each another, rearrange, and they are characterized by instability [2, 3]. An assumption was made back in the 1930s that these differences are caused by the presence of specific nucleotide sequences at the chromosome ends; these sequences were referred to as telomeres [3–5]. The telomeres consist of repeating sequences and a set of special proteins, which interact with these repeats and spatially organize them in a specific manner, resulting in the formation of the nucleoprotein complex known as telomeric heterochromatin [6, 7]. Shortening of the 5’-terminus of the daughter strand, caused by the removal of the terminal RNA-primer and the subsequent incomplete replication of linear DNA molecules, is observed during the genome replication occurring upon cell fission. The independent formulation of the so-called “end-replication problem” was proposed in the 1970s by A.M. Olovnikov and J. Watson [8, 9]. Olovnikov hypothesised that there is a special enzyme, i.e. telomerase, which is capable of compensating for the “end-replication problem.” This enzyme was discovered in 1987 by C. Greider and E. Blackburn [10]. 

Telomerase consists of two major components: reverse transcriptase (TERT) and telomerase RNA (TER), which contains the template domain for the telomere repeat synthesis [9]. Furthermore, the telomerase complex contains numerous additional components that ensure the *in vivo* activity of the enzyme. Additional proteins participate in various processes. A number of these proteins are required in order for telomerase to attach to a telomere at a certain instant of the cell cycle [10], whereas the others serve to regulate the enzymatic activity [11]. It is already known that telomerase does not function in all higher eukaryotic cells; however, its components are almost always present in a cell. The data on the non-telomeric functions of telomerase components were recently reported. 

## TELOMERE STRUCTURE 

**Fig. 1:**
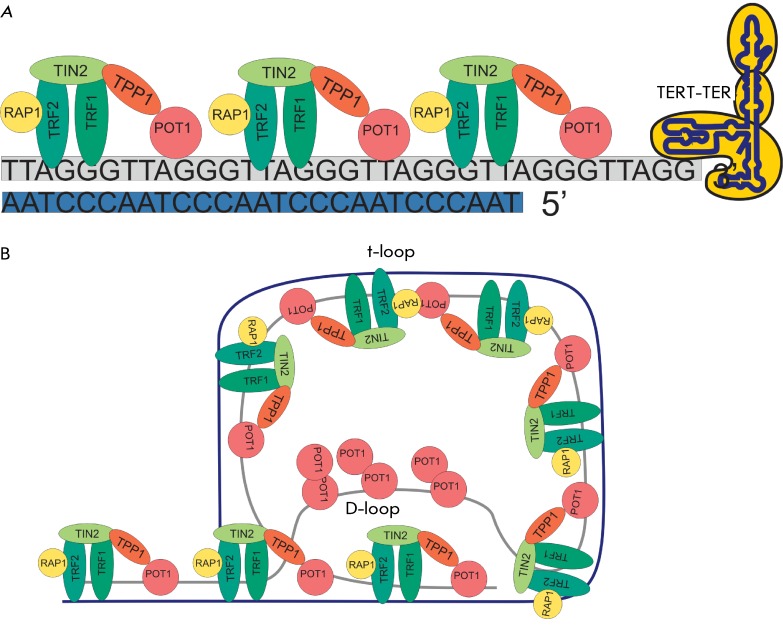
Telomere structure. *A* – Schematic representation of the telomeric DNA complex, proteins of the shelterin complex and telomerase. *B* – Schematic representation of the shelterin complex bound to telomeric DNA, in the t-loop conformation.

Telomeres are the repeating nucleotide sequences bound to the specific proteins protecting chromosome ends against degradation and the double-strand break repair systems [12, 13]. As data accumulated, a hypothesis was postulated that telomeres consist of three distinct regions. Firstly, they contain the so-called cap, a terminal structure protecting the chromosome ends against degradation and the double-strand break repair systems (DDR – DNA damage response); they also regulate telomere elongation. The major component of a telomere is a double-stranded DNA (dsDNA) consisting of repeating and transcribed sequences. The third component of a telomere is represented by repeating telomere-associated sequences, the so-called subtelomeric regions [14, 15]. The telomere nucleotide sequence is enriched in thymidine and guanosine residues and is appreciably conserved. Mammalian telomeres are a double-stranded region consisting of TTAGGG repeats and the 150–200 nucleotide long 3’ G-strand overhang. According to one of the hypotheses, the G-strand overhang is intertwined with the double-stranded telomeric region, thereby forming a t-loop. The so-called D-loop is formed at the site of the interaction between the protruding 3’-terminus with the double-stranded region ( *Fig. 1* ). t-Loops were detected via electron microscopy after DNA was extracted and treated in a special manner. However, the existence of these structures in cells has as yet not been unequivocally proven; therefore, the D-loops are considered as tentative structures. Telomere functions depend on the minimal length of telomeric repeats and the activity of the protein complex associated with them. This complex is known as shelterin and consists of six proteins: TRF1, TRF2, POT1, TIN2, TPP1, and RAP1. The proteins TRF1, TRF2 (telomeric repeat binding factor 1 and 2) and POT1 (protection of telomeres protein 1) are bound to telomeric DNA. TRF1 and TRF2 are bound to the double-strand telomeric regions; РОТ1 can be bound to the 3’-protruding single-stranded region of the G-strand [16]. TRF1 and TRF2 bind telomeres independently; they do not interact with each other. Both proteins, which have the form of a homodimer and an oligomer, are capable of specifically binding the DNA duplex to the telomeric sequence 5’-YTAGGGTTR-3’ [16–20]. POT1 binds highly specifically to the telomeric single-stranded DNA (ssDNA) 5'-TAGGGTTAG-3', attesting to a possible interaction both with the G-strand overhang and with the sequence of the D-loop displaced by it [13, 21–23]. POT1 interacts with TRF1. It is believed that TRF1 facilitates the binding of РОТ1 to the single-stranded telomeric region in this manner. Via its independent domains, TIN2 (TRF1-interacting protein 2) simultaneously interacts with TRF1 and TRF2, as well as with the ТРР1–РОТ1 complex, forming a bridge between the shelterin components [24, 25]. The C-terminal domain of TPP1 is bound to TIN2, the central domain is bound to POT1 [26–29]; thus, POT1 is attracted to the telomeres [30, 31]. Moreover, TPP1 contains a domain interacting with telomerase on its end. This fact supports the assumption that TPP1 attracts telomerase to the chromosome end [32]. Protein RAP1 forms a complex with TRF2 and the telomere [33, 34]. It has been demonstrated in studies undertaken by several research teams that RAP1 is not essential for telomere capping; however, it impedes recombination at telomeric regions and enhances their stability [35, 36]. Thus, RAP1 (unlike TRF1, TRF2, POT1, and TPP1) does not protect telomeres [32, 35, 36]. 

There is a hypothesis holding that G-quadruplex structures are formed in the telomeric regions of chromosomes. Four telomeric repeats can form a G-quadruplex, which inhibits telomerase activity [37–41]. The formation of these structures in ciliate cells has been clearly demonstrated using G-quadruplex-specific antibodies [42, 43]. The ability of the telomeric regions of higher eukaryotes to form G-quadruplex structures was indirectly supported by experimental data. According to [44], long 3’-protruding telomere ends form the G-quadruplex *in vitro* . The ligands binding the G-quadruplex structures are known to cause telomere shortening in cells. The telomerase activity remains intact, but the interaction between the shelterin complex and telomeric DNA is disrupted. Telomeres become instable, and their binding to POT1 is disrupted, resulting in the activation of the DNA damage response system. This may be an indicator of the adverse effect of the stabilization of the G-quadruplexes in telomeric regions. These structures can presumably form in transition states; however, the telomeres cannot permanently maintain the structure of G-quadruplexes [45]. 

The chromosomes in eukaryotic cells are known to be packaged into chromatin by special proteins. It is believed that chromatin in a condensed state is untranscribed: euchromatin being associated with the cell transcription apparatus [46]. The telomeric regions of chromosomes form the so-called telomeric chromatin [47]. The assumption has been made that telomere elongation can depend upon the epigenetic status of telomeric chromatin [48]. Both the telomeric and subtelomeric regions are known to be enriched in histones that are typically bound to the repressed heterochromatic regions, such as histones Н3 (Н3К9m3) and Н4 (Н4К20) trimethylated at lysine 9 and lysine 20 residues, respectively. Heterochromatin binding proteins1α, 1β, and 1γ (known as СВХ5, СВХ1, and СВХ3, respectively) also bind to these regions [49–51]. Moreover, it has been ascertained that telomeric DNA is strongly methylated. The telomeres in the chromosomes in cells without N-methyltransferases (SUV420H1 and SUV39H1), which modify lysine residues in histones, are too long [49, 50]. The same was observed in cells with reduced methylation status of subtelomeric regions due to the deficiency in DICER1 or DNA-(cytosine-5)methyltransferases 1, 3A, and 3B (DNMT1, 3A and 3В) [52]. RNA containing telomeric repeats (TERRA – Telomeric Repeat containing RNA), or telomeric RNA – TelRNA, which is formed as a result of telomere transcription, was recently detected. These RNAs are capable of interacting with telomeric chromatin and of *in vitro* suppression of telomere elongation by acting as a potential telomerase inhibitor [48, 53, 54]. One can reasonably assume that the synthesis of TERRA cells is repressed in oncotransformed cells, rendering them incapable of suppressing telomerase activity. 

Telomeric chromatin is dynamic, and its state may change. Differentiated somatic cells can be converted into induced pluripotent cells (iPS) via nuclear reprogramming [55]. The transition of the cells into a pluripotent state is accompanied by changes in the epigenetic status of telomeres: telomeric chromatin becomes less condensed; the histone content decreases, resulting in the subsequent formation of a large amount of TERRA; the level of telomere recombination becomes more frequent; and the telomere length becomes comparable with telomere length of embryonic stem cells [56]. Although no direct evidence exist so far to support the fact that the telomere length is regulated by changes in the chromatin state, the aforementioned observations lends credibility to the assumption that this theory is based on the truth. 

## TELOMERASE STRUCTURE 

The assembly of telomerase, its existence in a cell, and its entry to the telomeres are processes that are similar in some aspects, yet differ in other aspects with regards to evolutionary distant organisms [57–59]. Properties common to all telomerase components have been revealed: reverse transcriptase (TERT), telomerase RNA (TER), and TER-binding proteins, which stabilize RNA and facilitate the assembly of the active enzyme. It should be noted that only TERT is a highly conserved telomerase component. The data obtained through the study of the components within telomerase are rather inconsistent [60–64]. Telomerase apparently interacts with various components throughout its vital activity and thus can be found in various complexes. 

### TER structure 

**Fig. 2:**
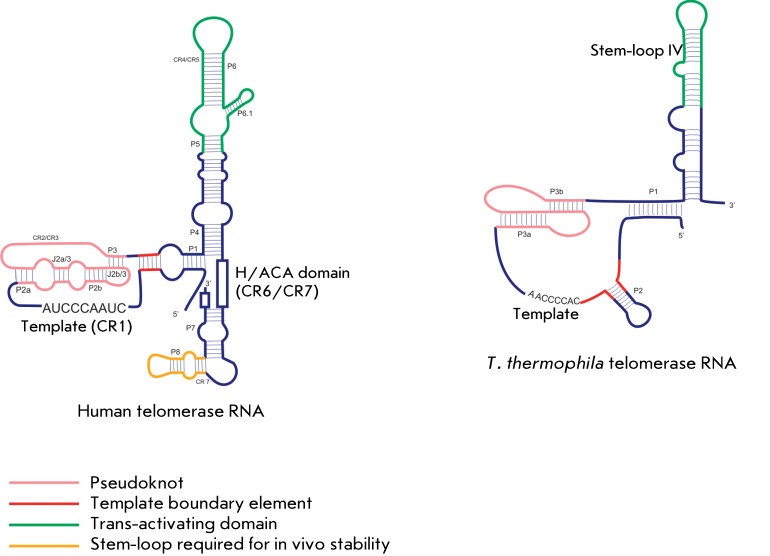
Structure of telomerase RNA. The schematic representation of the secondary structures of human and *T. thermophila* telomerase RNA. The conserved elements are shown in color.

Telomerase RNA is one of the major components of telomerase; it contains the region that acts as a template for telomere synthesis [65, 66]. Despite the differences in length and the nucleotide sequence of telomerase RNAs derived from different organisms, secondary structures of TER demonstrate high levels of similarity and contain similar structural elements [65, 67]. The template region, the pseudoknot, the trans-activating domain, and the domains required to ensure *in vivo* stability are the conserved elements of the TER structure ( *Fig. 2* ). Thus, TER contains the elements that are essential for telomerase activity and for the assembly, localization, and stability of RNA, but it is not required itself for enzymatic activity. The template domain of TER interacts with the 3’ G-strand overhang of telomeres and guides DNA synthesis. This region can be single-stranded, although the differences in structure were detected via the analysis of the secondary structure of the transcript obtained *in vitro* and TER carried out in the *in vivo * experiments, attesting to its interaction with the other cell components [68–70]. Data indicating that a triplex structure is formed between the pseudoknot elements and the template domain was recently obtained by NMR spectroscopy. It is presumably the formation of this structure that can explain the differences in the structure of the template domain of TER [71]. A hypothesis has also been put forth that in the absence of TERT and other necessary components, TER cannot form the correct structure. The template domain is flanked by two elements: the 5’-template boundary and the 3’-template recognition ones [72–75]. The 5’-element is a double-stranded region located immediately before the template domain; it regulates nucleotide addition during reverse transcription and, presumably, is the binding site with TERT. It has been demonstrated using mutagenesis that it is the secondary structure of this region, rather than the nucleotide sequence, that is of significance for the efficient functioning of telomerase. The 3’-recognition element is a single-stranded structure located after the template domain, which allows the 3’-terminus of the template to occupy the active site, stimulates telomerase activity and the ability to process after the repeats are added, and it contains the binding site of the N-terminus of TERT [76, 77]. 

Among the elements of the secondary structure of telomerase RNA, the pseudoknot has been the most intensely studied element. Changes in the stability of the pseudoknot result in a reduction in telomerase activity, which attests to the fact that this structural element plays an essential biological role [78, 79]. The recent study of oligonucleotides representing the structural elements of the TER pseudoknot via NMR spectroscopy and molecular modeling have proved that it is the dynamics of the tertiary structure of the pseudoknot that plays a significant role in telomerase functioning [80–85]. The pseudoknot is formed due to the formation of the evolutionary conserved Hoogsteen triplet U*A*U between the U-rich loop 1 (J2b/3) and the major stem-loop (P3), which facilitates the maintenance of the structural integrity and is required for telomerase activity. Meanwhile, the A-rich loop 2 (part of J2a/3) enters the two other non-canonical triplet interactions, which facilitate the stabilization of the pseudoknot [86, 87]. Another Hoogsteen pair, A*U, is located between these two structural elements consisting of triplets. This pair is responsible for the stacking interaction between the two main stems, resulting in the formation of the final structure of the triple helix [85]. Nucleotide mutations inside the pseudoknot result in disintegration of the tertiary structure and considerably reduce telomerase activity, whereas compensatory mutations restore telomerase activity. These data confirm the fact that the tertiary structure has a more significant impact on the catalytic activity of the enzyme, compared to that of the nucleotide sequence [71, 87, 88]. It was assumed that the pseudoknot is necessary for correct orientation the template-primer duplex at the telomerase active site [71]. It is possible that the ability of this structure to exist in two conformations, the pseudoknot and the stem-loop, is of significance for telomerase functioning [83]. 

Unlike the pseudoknot, the structure of the trans-activating domain of TER has been subjected to less thorough study. The primary structures of this domain derived from different organisms are characterized by a high level of homology [7, 9, 87]. The trans-activating domain is a long stem-loop consisting of several, extremely stable helices broken apart by asymmetric loops and single-nucleotide bulges. This domain is required for the correct formation of the pseudoknot, nucleotide addition, and telomerase processivity upon repeat addition [88–90]. The P6.1 helix of the trans-activating domain of human TER is the one that has been best studied. This element is essential for enzyme functioning [90–93]. The role of the P6.1 in vertebrates has not been completely elucidated; however, it has been known that accurate structure of this helix is necessary for telomerase assembly, whereas specific sequences in the loops play a significant role in catalysis [93]. It is believed that the interaction of the P6.1 loop with the template domain yields the tertiary structure of TER, which thus explains the role of these enzymes in telomerase activity and ability to process [94]. 

The H/ACA domain, which is present in small nucleolar RNAs (snoRNAs) and in small Cajal body specific RNAs (scaRNAs), is located at the 3'-terminus of TER in vertebrates. The H/ACA domain is a single-stranded region containing the H-box (ANANNA, where N is a random nucleotide), the stem-loop that follows (containing the CAB-box), and the single-stranded 3’-terminus containing the ACA-box [94, 95]. The H/ACA domain is required to ensure the *in vivo* stability of telomerase RNA [96]. The CAB-box acting as a signal of localization in Cajal bodies is located inside this domain. The CAB-box does not participate in the 3’-terminal processing of telomerase RNA [97]. 

Data attesting to the fact that the first 17 nucleotides of human TER are essential for telomerase activity, and that absence of this region or mutation in it considerably reduces the enzymatic activity, have recently been reported. The ribooligonucleotide with this sequence was shown to form a G-quadruplex. It can be assumed that the structure of this element is likely to affect the structure of the P1 helix and positioning of the template domain of telomerase RNA [98–100]. 

### Structure of telomerase reverse transcriptase 

The telomerase catalytic subunit, TERT, is much more conserved in comparison with TER. It has a large number of motifs that are common to the other reverse transcriptases. Three domains can be distinguished in the structure of all known TERT: the RNA-binding domain (also known as TRBD and subdivided into RID1 and RID2), reverse transcriptase domain, and the poorly conserved C-terminal domain [57, 95, 101]. Certain TERT contain an additional N-terminal TEN domain, which is involved within the process of primer binding and facilitates the processive addition of telomere repeats [57, 102]. The primary structure of the reverse transcriptase domain is similar to the structures of ot­her polymerases and contains seven conserved motifs (1, 2, А, В, С, D, and Е). It is assumed that TERT originates from retrotransposons [103]. Intron-containing (the so-called Penelope-like) elements are the ones most similar to TERT. 

**Fig. 3:**
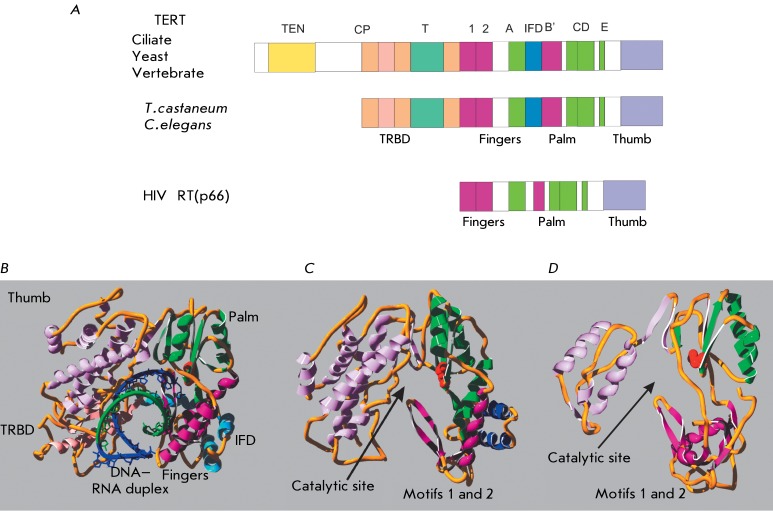
Structure of telomerase reverse transcriptase. *A* – Schematic representation of the domain organization of TERT in different organisms and HIV reverse transcriptase. Homologous domains are shown in color. *B* – Spatial structure of the *T. castaneum* TERT complex and the RNA–DNA duplex. The image was obtained using the PDB file 3KY. *C* – Spatial structure of *T. castaneum* TERT. The image was obtained using the PDB file 3DU5. *D* – Spatial structure of HIV RT. The image was obtained using the PDB file 1N6Q. Asn residues of the enzyme catalytic sites are shown in red.

Structures with high-resolution TEN and TRBD TERT domains and full-length TERT were recently derived from *Tetrahymena thermophila* [102, 103] and *Tribolium castaneum* [104], respectively; they showed new features of telomerase structure and function. As follows from the analysis of the structure of TERT derived from *T. castaneum, * high structural homology exists between TERT and other polymerases, including the p66 subunit of HIV reverse transcriptase [105]. The spatial arrangement of the major domains and of the key amino acid residues in them remains constant and corresponds to the right-hand structure that was first described for the Klenow fragment of DNA polymerase I from *Escherichia coli* . It is common to distinguish the so-called palm, fingers, and thumb subdomains in these structures [106]. It was revealed that the loop responsible for the binding and positioning of the template and nucleotide is located between the β-sheets of motifs 1 and 2 and oriented towards the active site [107, 108]. Meanwhile, there also are differences in the structure of TERT and other polymerases ( *Fig. 3* ). Thus, TERT contains an additional domain known as IFD (insertion in fingers domain). This domain is located outside the central ring between the “fingers” and the “palm.” It is clear from the structure of TERT derived from *T. castaneum * that three completely conserved domains form a ring-like structure. The conserved reverse transcriptase domain forms “fingers” and a “palm” (similar to the other polymerases) and occupies one side of the ring, whereas the C-domain forms the “finger” and is located in direct proximity to the N-terminal RNA-binding domain and thus closes the ring. The inner diameter of this structure is 26 Å, and its depth is 21Å; approximately corresponding to the size of the A-helix consisting of 8 bp. The DNA–RNA duplex located in the polymerase active site has precisely this type of structure [109]. The surface of the opening is a spiral structure consisting of positively charged amino acid residues. This structure allows the protein to make close contact with the RNA–DNA heteroduplex [110]. The helices 10 and 19 interact with the major and minor grooves of the DNA–RNA duplex located in the active site [111–115]. The nucleotide binding domain is located at the “fingers”–”palm” interface; this fact is attested to by the high levels of similarity with the other polymerases. Several conserved amino acid residues forming the nucleotide-binding pocket have been determined [115]. The active site of the enzyme contains several unchangeable aspartic acid residues and a lysine residue, which activates the pyrophosphate leaving group. 

As follows from an analysis of its structure, the TRBD domain of *T. thermophila* and *T. castaneum * TERT is enriched in helical structures and is divided into two parts. These two structural elements are bound via CP- and T-motifs. The CP-motif contains a positively charged pocket, whereas the T-motif is a narrow hydrophobic gap with positively charged residues near the CP-motif. Together, they form an extensive groove on the surface of TRBD, to which TER is bound [100, 103, 116–119]. The T-motif contains a β-stem-loop stretched in the direction of the C-terminal domain, which forms the “thumb,” and links it to the “fingers” of the reverse transcriptase domain. This arrangement of the TRBD domain allows the residues located on the inner side of the ring to come in direct proximity with the active site. Moreover, the gap between the TRBD and reverse transcriptase domains enables TER to get into the active site. One can assume that telomerase RNA penetrates through this gap, thus carrying the template into the enzyme’s active site [120]. 

## TELOMERASE BIOGENESIS 

As previously mentioned, telomerase consists of the two major components; however, the synthesis and processing of each component, as well as the formation of an active enzyme, require the contribution of a large number of additional factors. The regulation of TERT expression at the transcriptional phase was thoroughly discussed in the review by Skvortsov *et al* . [121]. The alternative splicing of the primary transcript of the *hTERT* gene yields 13 different mRNA variants [122–125]. Out of these variants, the so-called α- and β-forms are both the most common and well-studied. When the α-form is produced, 36 nucleotides are deleted from the sixth exon, resulting in the change of the reverse transcriptase motif A. The open reading frame is not disrupted [126, 127]. The deletion of 182 nucleotides from the exons 7 and 8 and the insertion of 38 nucleotides triggers a premature translation termination, resulting in the formation of the β-hTERT, which does not contain the three essential reverse transcriptase motifs [128, 129]. Splicing can independently occur at different sites; therefore, different forms of *hTERT* mRNA often co-exist in cells. The combination of different mRNA forms and their number depend on the particular cell type. Thus, one of the mRNA variants (α-/β+ form) has regulatory functions by acting as a dominant negative inhibitor of telomerase activity both in normal and tumor cells. 

It remains unclear as to whether the ratio between the full-length *hTERT* mRNA and its spliced forms affects telomerase activity. The total level of *hTERT* expression was shown to correspond to the level of telomerase activity in some studies [126–129], whereas no regularities in the alteration of telomerase activity with the variation of any *hTERT* mRNA form have been revealed [130]. It is assumed that the regulatory functions executed by the products of the alternative splicing of *hTERT* are cell-type dependent. The set of *hTERT* transcripts changes during human embryonic development. During the early stages, all tissues contain full-length *hTERT* mRNA and active telomerase; subsequently, the set of mRNA forms changes depending on a tissue’s type [131]. One can assume that the variation of TERT expression is required for cell differentiation during the development of the organism. 

Reversible phosphorylation of TERT plays a significant role in the regulation of the telomerase function [132]. Numerous kinases and phosphatases are already known. They affect the phosphorylation of serine, threonine, and tyrosine residues, thus changing the structure, localization, and activity of enzymes. Non-specific phosphorylation sites have been detected in the primary structure of *hTERT* ; however, only a few of these can be modified, and their phosphorylation affects telo­merase activity. 

Telomerase RNA belongs to the family of non-coding RNAs; i.e., it does not act as a template for protein synthesis. As mentioned above, telomerase RNA contains structural elements that are characteristic both to small nucleolar RNAs and to small Cajal body specific RNAs. All human Н/АСА RNAs are encoded by introns, which are synthesized in the form of pre-mRNA and are processed to yield mature RNAs without the cap structure at their 5’-terminus [133]. In contrast, human TER is transcribed by RNA polymerase II from its own promoter. The processing of the primary transcript results in the formation of the 451 nucleotide mature form containing a trimethylguanine cap at its 5’-terminus. The processing of telomerase RNA has been partially studied in yeast cells. It is known that the 3’-terminal processing of TER in * Schizosaccharomyces pombe * cells is performed by a spliceosome. Only the first splicing stage resulting in the release of the 5’-terminal exon is required for the formation of active telomerase RNA [134]. Exon ligation would yield the rapidly degradable product. It is unclear how the splicing is terminated at the intermediate stage. 

Trimethylation of 5’-terminal guanine in TER in yeast cells is performed by methyltransferase Tgs1. It is assumed that in vertebrates this enzyme (hTgs1р) also participates in the hypermethylation of the 5’-cap of TER in Cajal bodies, in which it is contained [135]. 

The study of the processing of human telomerase RNA is complicated by its low content in cells. Human telomerase RNA is expressed and undergoes processing in yeast cells [136]. Both polyadenylated and non-polyadenylated, processed and non-processed hTER forms are produced during expression in yeast cells. Processing of hTER in this system is performed by yeast proteins Cbf5p (dyskerin homolog), Nhp2p, and Nop10p, which participate in the processing of small nucleolar RNAs containing the H/ACA-domain [137–139]. H/ACA-proteins are bound to the H/ACA-domain of telomerase RNA, which determines the 3’-boundary of the mature hTER. It is assumed that during the processing of hTER, its 3’-terminus is cleaved via exonucleases, whereas the H/ACA-proteins that have been bound determine the boundary of the mature hTER form [136]. A hypothesis that nucleases are activated as a result of H/ACA-protein binding to telomerase RNA has also been postulated [140]. 

It was ascertained in 2011 that the telomerase complex contains the DHX36 protein, or RHAU (known as RNA helicase). It also participates in the degradation of mRNAs containing AU-rich elements and is the resolvase both for the DNA and RNA of G-quadruplexes [141–145]. It was found that this protein interacts with the 5’-terminal region of the hTER forming the G-quadruplex and stabilizes hTER. This occurs before the 5’-terminal guanosine is trimethylated by telomerase RNA and is presumably required to protect hTER against degradation. Once hTER is capped, the formation of the G-quadruplex is no longer feasible and RHAU is no longer able to bind to hTER [146]. Furthermore, RHAU stimulates the formation of helix P1,thereby providing the correct positioning of the template domain of hTER [147]. 

The telomerase complex contains additional proteins, which participate in enzyme biogenesis [148]. The telomerase complex always contains the RNA-binding protein dyskerin, which is capable of recognizing the H/ACA-motif both in telomerase and in the other non-coding RNAs (small nucleolar and Cajal body specific RNAs) [149, 150]. Dyskerin is believed to participate in the biogenesis of telomerase RNP (ribonucleoprotein) and maintains the stability of telomerase RNA [151, 152]. The telomerase complex may contain the dyskerin-binding proteins NOP10, NHP2, and GAR1 [153, 154]. DNA helicases pontin and reptin, which exhibit ATPase activity, interact with hTER, hTERT, and dyskerin [155]. The content of the hTERT complex with these proteins is highest in the S-phase of the cell cycle. Telomere elongation in yeasts occurs precisely at this moment [156], whereas in human cells telomerase is associated with telomeres [157, 158]. It can be reasonably assumed that pontin and reptin can affect the regulation of the hTERT content at different phases of the cell cycle, or affect the assembly of active telomerase in the S-phase. Dyskerin is permanently bound to hTER, whereas pontin and reptin interact with hTERT. In the S-phase, pontin and reptin interact with dyskerin by participating in a *de novo* formation of the telomerase RNP. 

One of the recent studies is devoted to the identification of another protein participating in the assembly and effecting of telomerase activity [159]. It was shown that this protein is ATPase NVL2. It was de­monstrated using the two-hybrid system that hTERT interacts with the NVL2 protein. The *NVL * gene encodes two isoforms of NVL ATPase (NVL1 and NVL2), which belong to the AAA (ATPase associated with a variety of cellular activities) family of ATPases [160, 161]. hTERT interacts with both isoforms; however, the complex with NVL2 turned out to be stronger. In cells, hTERT is co-localized with NVL2, which contains two ATPase domains. The Lys311 mutation in the first domain disrupts the binding of this protein to hTERT, whereas NVL2 knockdown reduces the telomerase activity in cells [159]. 

The telomerase holoenzyme contains the WDR79/TCAB1 protein (telomerase Cajal body protein 1) [58]. Cajal bodies are enriched in this protein, which is associated with TERT, TER, and dyskerin. Meanwhile, ТСАВ1 does not interact with the telomerase assembly factors NAF1, pontin and reptin. It is assumed that pontin and reptin at the first stage of telomerase maturation facilitate assembly of the minimally active enzyme consisting of TERT, TERC, and dyskerin. TCAB1 subsequently interacts with active telomerase and determines its localization in Cajal bodies, thus facilitating the binding to telomeres. 

The data regarding the architecture of the telo­merase holoenzyme lack consistency. Thus, telomerase has been demonstrated to possess catalytic activity only when in dimeric form; however, it has also been claimed that dimerization is not a prerequisite under physiological conditions [162–164]. The immunoprecipitation method was used to study the composition of the proteins isolated along with telomerase [165]. It turned out that telomerase can form several comple­xes differing by the proteins they consist of. It was assumed that the composition of the telomerase complex changes during maturation. At the first stage, the H/ACA-proteins are bound to the 3’-terminal stem-loop of hTER; the second complex of H/ACA-proteins with the GAR1 protein subsequently interacts with the stem of the СR4/CR5 stem-loop. At the second stage, GAR1 is replaced from the telomerase RNP by the ТСАВ1 protein and is bound to hTERT. Meanwhile, TERT and TCAB1 are present in the complex at a substoichiometrical ratio. This fact may be evidence of the existence of either a single complex containing both components, or two different complexes, each of those containing one of the components. It was assumed that both complexes are simultaneously present in cells; however, they permanently exchange components. 

The interaction between telomerase and the other proteins (La, Staufen, L22, hnRNP, C1/C2, TEP1, p23, and Hsp90) is required for the formation of the appropriate structure and its stabilization [132, 166, 167]. However, it is unclear whether these proteins affect the telomerase activity. 

## TELOMERE ELONGATION 

The major activity of telomerase ensures the RNA-dependent telomere elongation [168]. The telomerase catalytic cycle consists of several sequential stages. One telomeric repeat is added after the substrate binding. The resulting product can either dissociate from the enzyme’s active site or undergo translocation, followed by elongation. The ability of telomerase to move the DNA synthesized to the template beginning site allows one to use two processivity types to describe its function. Nucleotide addition (type I processivity) is intrinsic to all polymerases, since repeat addition (type II processivity) is unique to telomerase and determines the ability of an enzyme to repeatedly copy an RNA template region via elongation of the one substrate molecule only [169, 170]. 

Primer binding at the first stage of the telomerase reaction cycle is stipulated by its complementary action with the TER template region. When using primers with different sequences, the efficiency of formation of the complex with an enzyme does not correlate with the length of the resulting DNA–RNA duplex [171], since telomerase is bound to the substrate upon immediate participation of not only the template region of telo­merase RNA. The structural elements of the TERT active site regulate the efficiency of duplex formation, as well as translocation of the freshly synthesized product during the processive synthesis of telomeric repeats. The anchor regions in TERT and TER also participate in the primary binding of the primer. 

Nucleotides are bound to the primer at the second stage of the telomerase reaction cycle [168, 172]. 

The major feature of telomerase is its ability to processively add the repeats [170]. The mechanism of telomerase translocation after a repeat is synthesized remains unknown. It remains ambiguous as to whether enzyme processivity of this type is required for efficient telomere elongation or not. It has recently been ascertained that critically short telomeres elongate processively [173]. A set of products differ in the number of telomeric repeats is formed during telomerase operation. After a single telomeric repeat is added, the reaction is either terminated or the rate of reaction decreases; i.e., template translocation and annealing are the rate-limiting stages. It has been demonstrated that POT1 and TPP1 proteins efficiently stimulate telomerase processivity [174]. An assumption was made that telomerase processivity is regulated by the POT1–TPP1 complex. Telomerase activity is inhibited when the complex is bound to the 3’-terminus of the primer. When it is bound to the 5’-terminus, telomerase functions processively. 

It is known that telomerase is not active in all cells. Nevertheless, telomerase RNA occurs in all cells; reverse transcriptase occurs in the majority of cells. The localization of telomerase components does not necessarily coincide with the site of its “operation.” Telomerase RNA frequently occurs in the cytoplasm; reverse transcriptase is found in mitochondria and other organelles. These data enable to assume that telomerase can have additional functions in the cell rather than just maintenance of the telomere length. 

## ALTERNATIVE FUNCTIONS OF TELOMERASE COMPONENTS 

**Fig. 4:**
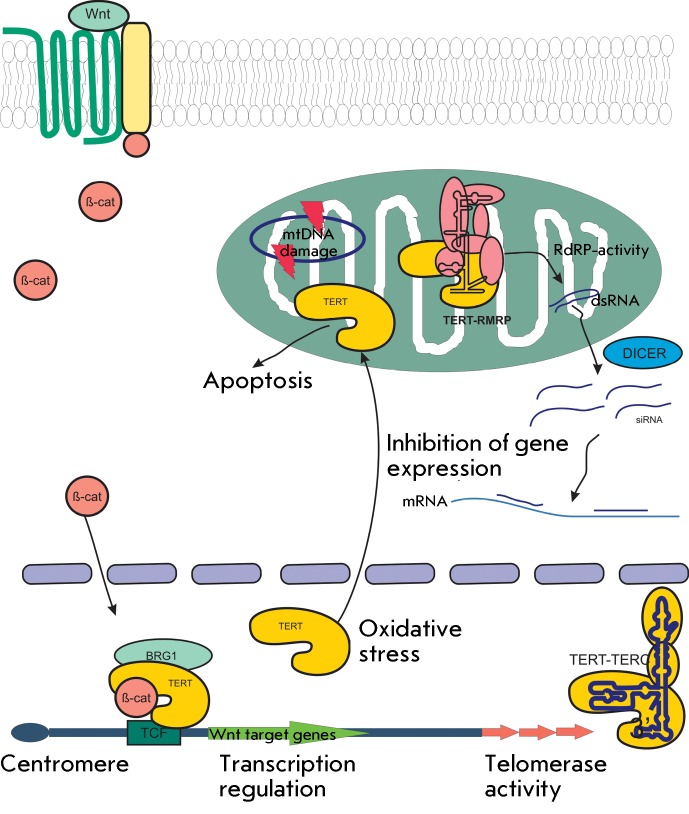
Telomerase is the enzyme with versatile functions. Schematic representation of the mechanisms of telome­rase functions in the cell.

The first batch of data on the alternative functions of telomerase were reported at the early stages of the study of this enzyme. The products of other enzyme activities were detected when studying the activity, substrate specificity, and other properties of telome­rase. It turned out that telomerase is also capable of acting as a catalyst for the other reactions ( *Fig. 4* ). 

### Telomerase nuclease activity 

It was demonstrated through a study of the catalytic activity and substrate specificity of telomerase derived from *Thermus thermophila * that the length of the resulting product depends on the complementarity degree of a primer and the template region of te­lomerase RNA [175]. If the 3’ terminus of the primer is non-complementary to the template region, a break at the boundary between the coupled and non-coupled substrate regions occurs. Moreover, the break is also possible in a entirely complementary primer. In this case, the site and possibility of a break will depend on the length and site of preferential primer annealing at the template region. Thus, telomerase derived from *Th. thermophila * exhibits nuclease activity. It was later ascertained that human and yeast telomerases also exhibit such activity [176–181]. A thorough investigation into the mechanism of endonuclease activity has de­monstrated that a substrate can be cleaved even when it is completely complementary to the template so that it could be preferentially located in the telomerase catalytic site. The endonuclease activity of telomerase is not sequence-specific. The primers having non-hydrolyzable internucleotide bonds at preferential cleavage sites are subjected to cleavage at other sites [179, 182]. 

### Transferase activity 

Yeast and human telomerase can also exhibit transferase activity. In the presence of Mn ^2+^ -ions, telomerase can add nucleotides independently of the template. Preference is given to telomere-like primers with GT-rich 5’-end. It is unknown whether there can be *in vivo* situations when the intracellular Mn ^2+^ concentration reaches values at which transferase activity is detected *in vitro* . Nevertheless, it has been assumed that certain small molecules can stimulate this ability of telomerase [183, 184]. 

### Telomerase and mitochondria 

It was revealed during the early stages of the investigations into telomerase that hTERT is also expressed in cells where telomerase activity is not detected [185, 186]. It has recently been reported that the hTERT protein occurs in each somatic cells, predominantly in the S-phase [187]; moreover, it is present not only in the nucleus, but it is also present in cytoplasm and mitochondria [188–194]. In immortalized human fibroblasts under treatment with Н _2_ О _2 _ and oxidative stress conditions, hTERT is exported from the nucleus and transferred to the mitochondria [194]. Mouse embryonic fibroblasts are known to undergo earlier senescence if cultivated in conditions with increased oxygen content [195]. As shown in similar experiments using human cells, oxygen deficiency results in an increased life span [196, 197]. The oxidative stress can activate the tumor suppressor proteins p53 and Rb [198, 199]. There are several factors that cause cellular senescence under oxidative stress conditions. Firstly, they include DNA damages effected upon oxidative stress and induce the activity of the regulators of the cell cycle р21 and р16, which facilitate cellular senescence and cell cycle delay [200, 201]. As a result of decreased proliferation, telomeres critically shorten; subsequently, the cells experience a crisis. At this stage, the cells either die, or telomerase is activated, making them immortal. Secondly, oxidative stress can cause damage of telomere. The DNA within the telomeres is enriched in guanine residues that are sensitive to oxidation. Due to the oxidation of these residues telomeres become more sensitive to damage, their degree of shortening increasing [202, 203]. It has been demonstrated that the treatment of cells with mitochondria-targeted antioxidant MitoQ reduces the level of telomere damage and prolongs the life span of the fibroblasts subjected to oxidative stress [204]. Thirdly, premature senescence induced by oxidative stress may be a result of the direct inactivation of telomerase activity. 

It was predicted recently that the N-terminus of TERT contains the mitochondria transport signal (MTS) consisting of 20 amino acid residues [205]. MTS is highly conserved in the TERT of higher eukaryotes, such as plants, fish, and mammals; however, yeasts and ciliate organisms contain none of this sequence [192]. The green fluorescent protein (GFP) with MTS localizes in mitochondria. The *in vitro* synthesized protein A containing MTS from hTERT at its N-terminus is transported into the purified mitochondria through the membrane potential [205]. Various methods have been used to detect the localization of hTERT in mitochondria, including immunoblotting and coimmunoprecipitation [191, 192, 194, 206–208]. 

Mytochondrial extracts from various human cells exhibit telomerase activity. hTERT is located in the mitochondrial matrix and is co-precipitated with the proteins ТОМ20, ТОМ40, and TIM23 [191]. It has also been demonstrated that hTERT is extracted along with the mtDNA-binding proteins TFAM, HSP60, and TIM23, but not with TOM20 [205]. Increased hTERT content in mitochondria subjected to oxidative stress results in the stabilization of mtDNA and stimulates the mitochondrial function. This suppresses the formation of reactive oxygen species and increases the potential of the mitochondrial membrane [194]. hTERT interacts with the mtDNA regions encoding the subunits 1 and 2 of the NADH–ubiquinone oxidoreductase (ND1 и ND2). Changes in respiratory chain activity were observed in mouse cardiac (but not hepatic) cells expressing hTERT [191]. Nevertheless, non-specific interaction between hTERT and mtDNA was later detected[205]. The chromatin immunoprecipitation assay modified for mitochondria was used to demonstrate that telomerase reverse transcriptase interacts both with the regions encoding ND1, ND2 and with 12S and 16S rRNA, ND4 and ND5, COXI and COXIII, tРНК and the subunits 6 and 8 of АТР-synthase [205]. It is known that increased expression of hTERT in human fibroblasts does not prevent the stress-induced senescence, but protects them against apoptosis and necrosis [209]. The opposite effect of increased expression of hTERT under oxidative stress (i.e., increased degree of DNA damage) has also been demonstrated [192, 206]. The content of a bio-accessible iron, which can stimulate the formation of the hydroxyl radicals that damage DNA, increases in these cells [192]. 

The factors responsible for the intracellular transport of hTERT remain unknown. hTERT contains the mitochondria transport signal and the nuclear export signal (NES). It has been hypothesized that hTERT contains several nuclear localization signals (NLS) [210, 211]. However, the regulation mechanism of the localization of this protein inside the cell remains unclear. It is a known fact that the intracellular distribution of hTERT changes under oxidative stress conditions due to posttranslational modifications [189, 194, 208, 210, 212–216]. Src kinase was shown to regulate the export of hTERT from the nucleus to the cytoplasm under oxidative stress [189, 194], whereas dephosphorylation of hTERT by phosphatase Shp-2 results in the import of hTERT from the cytoplasm into the nucleus [212]. During oxidative stress, Src kinase phosphorylates Tyr707 in TERT. The modified hTERT interacts with the nuclear pore component, protein Ran, followed by its export from the nucleus with the participation of karyopherin CRM1. Treatment of the cells with hydrogen peroxide reduces the level of wild-type mitochondrial hTERT, whereas the level of hTERT, in which Tyr707 is substituted by Phe and thus cannot be phosphorylated by Src kinase, remains unchanged. The mutant hTERTY707P expressed in cells is accumulated in the nucleus during the oxidative stress, the apoptosis level of these cells being lower than that of the cells containing the wild-type hTERT [189]. It has also been shown that treatment of cells with H _2_ O _2 _ results in an increase in the level of wild-type mitochondrial hTERT for several hours, whereas it takes several days for this effect to develop under hyperoxic conditions. hTERT returns to the nucleus in the cells that returned to the normal state after being cultured under hyperoxic conditions [194]. 

Several years ago it was revealed that hTERT can form complexes with the RNA-component of mitochondrial RNA that processes endoribonuclease RMRP, along with the complexes with hTER. It turned out that the hTERT–RMRP complex exhibits the activity of RNA-dependent RNA-polymerase and synthesizes double-stranded RNAs using the RNA-component of RMRP as a template. These RNAs are processed by the enzyme complex DICER yielding small interfering RNAs (siRNA), which subsequently reduce the RMRP cell level; i.e., the RMRP function is regulated according to the principle of negative drawback [217]. 

It has been reliably ascertained that hTERT functioning in mitochondria is hTER-independent, and hTER is not imported into mitochondria [205]. It turned out that mitochondrial tRNAs are released from the mitochondria, along with hTERT; the mitochondrial tRNAs act as primers in the reverse transcription reaction catalyzed by hTERT. Meanwhile, this reaction can be inhibited by the addition of hTER and mutation in one of the reverse transcriptase domains of hTERT. If the mitochondria contains no hTERT, the result is mitochondial dysfunction. It was assumed that hTERT can participate in the replication and repair of mtDNA [205]. 

It follows from the aforementioned facts that data regarding the functions of hTERT, and the components that interact with it in mitochondria, lack consistency. This fact can most likely be attributed to the limited amount of enzymes in a cell. All the studies devoted to the functions of telomerase in mitochondria have been carried out under conditions of protein overexpression. The additional domains that are used to extract and detect the protein can be the reason behind the unreliable results. It should be noted that at the time of writing, no clear-cut opinion has emerged within the research community regarding the functions of telomerase in mitochondria. 

### Telomerase and DNA damages 

Non-functional telomeres are known to interact with a set of proteins involved into the DNA damage response [218–220]. These proteins participate in signal transmission in response to different impacts. ATR and ATM belong to the family of protein kinases related to phosphoinositide 3-kinases (PIKK) [221]. ATM is the major protein activated on double-strand DNA breaks (DSB) [222], whereas ATR is activated when single-stranded DNA ends emerge upon the formation of DNA adducts, during DSB processing, or during termination of the replicative fork [223, 224]. The absence of ATM results in telomere decapping and shortening; furthermore, TRF2 is bound to ATM kinase to inhibit its activation [225].Suppression of ATR kinase activity is observed under conditions of increased expression of hTER. On the contrary, the decrease in the amount of telomerase RNA in cells facilitates an increase in ATR activity. These processes are independent from the level of telomerase activity and telomere length. A reduced level of hTER expression in cells results in an increase in the amount of protein p53, the tumor suppressor and the major contributor to the signalling pathway upon oncogenic stress. Meanwhile, the cell content of the protein CHK1, the cell cycle regulator, increases. p53 and CHK1 are the major substrates of ATR kinase. hTER inhibits ATR kinase and disrupts the regulation of the cell cycle checkpoints upon DNA damage *in vivo* [226]. 

The mutations in the template region of telomerase RNA induce a decrease in the level of the protein TRF2, which stimulates the apoptosis. This effect is ATM-dependent. ATM activation results in the phosphorylation of p53, which in turn activates the transcription of the *GADD45γ* gene. The increase in the GADD45γ level results in the apoptosis. Thus, mutations in the template region of telomerase RNA trigger DNA damage; the cells systems consider these damages to be double-strand breaks [227]. 

Histone H _2_ AX in eukaryotic cells is phosphorylated by ATM kinase in response to DNA damage. The phosphorylated γН _2_ АХ is bound to DNA at double-strand-break sites. In cells without hTERT, which were exposed to ionized radiation, the DDR system ceases to function. In fibroblasts with hTERT expression stably suppressed by RNA interference, the ATM and γН _2_ АХ content is reduced. The telomere length in these cells changes to a negligible degree, whereas the chromatin structure and post-translational modifications of histones are changed [187]. It is already known that the frequency of spontaneous chromosome breaks at the G1-phase decreases by 20-fold and the ATP level increases in human foreskin fibroblasts upon overexpression of hTERT [228]. The presumable reason is that hTERT has a protective role in mitochondria. ATP is required for the functioning of chromatin remodeling factors [229] and activation of ATM kinase [230]. Whilst protecting the mitochondria, hTERT presumably has a mediated effect on ATP synthesis in a cell, as well as on all the processes for which its hydrolysis is required. 

### Telomerase and regulation of gene expression 

The development of methods for the investigation of cell functioning and gene expression has enabled to study how the activity of some genes affects the expression of other genes. The cDNA microarray was used to ascertain that the expression level of 284 genes changes in bovine adrenal cells that overexpress TERT [231]. 

Nowadays, it is a known fact that telomerase can affect the cell cycle via regulation of the expression of various genes. An increase in the TERT level enhances the proliferative potential of human bone marrow stromal cells [232] and results in hyperplasia and hypertrophy of murine cardiomyocites [233]. The increase in the level of *hTERT* expression in human breast epithelial cells with the deleted *р16 * gene makes them resistant against the antiproliferative effect of the transforming factor β (TGF-β) [234]. Meanwhile, no dependence between the telomere length and cell sensitivity to TGF-β has been detected. It is also known that telomerase activation in human breast epithelial cells stimulates their processing into mitosis [235]. 

Evidence to the fact that telomerase affects the pRB/E2F signalling pathway has been obtained. Cyclin D upon mitotic stimulation form a complex with CDK4 and CDK6 and phosphorylate and simultaneously inactivate the retinoblastoma protein pRB. As a result, the interaction between pRB and the E2F transcription factor is disrupted. E2F is activated, triggering the expression of the genes required for cell transition from the G1- to the S-phase. Overexpression of hTERT in human crystalline lens cells induces their growth. Meanwhile, hyperphosphorylation of pRB and expression inhibition of р53, р21, and GCIP are observed [236]. р21 and GCIP are the inhibitors of cyclin complexes with cyclin-dependent kinases [237, 238], whereas p53 activates p21 transcription [239]. Thus, hTERT activates the pRB/E2F-dependent cell cycle pathway. On the other hand, hTERT stimulates the proliferation of human embryonic stem cells by shortening the G1-phase of the cell cycle [240]. This process is associated with the enhancement of the expression of cyclin D1 and hyperphosphorylation of pRB. One can assume that the transcriptional activity of E2F increases, since the level of one of its activators (CDC6) is increased. Moreover, pRB is hyperphosphorylated in hTERT-immortalized human foreskin fibroblasts and human adenoid epithelial cells, which overcome the crisis state after hTERT overexpression [241]. It is an interesting fact that in this case expression of p21 and p53 remains constant; furthermore, these cells contain no protein p16, which is an inhibitor of cyclin-dependent kinase CDK4/6 in complex with cyclin D [242]. 

Thus, hTERT-dependent stimulation of cell proliferation is induced by the inhibition of protein pRB and activation of the E2F transcription factor. Meanwhile, the same mechanism ensures apoptosis induction [243, 244]. 

It is known that hTERT overexpression in cells results in an increased content of the epidermal growth factor receptor (EGFR), the transmembrane receptor tyrosine kinase participating in the processes of growth, survival, proliferation, and differentiation of mammalian cells [235, 245]. Following ligand binding, EGFR becomes capable of activating different signalling pathways. Two of those (Ras/Raf/MEK/ERK and PI3K/Akt-kinases) participate in tumor development. These kinase cascades jointly stimulate cell entry into the S-phase of the cell cycle by affecting the expression, stability, and intracellular localization of D-type cyclins [246–250]. This fact is attested to by the results of experiments where the cells overexpressing the *hTERT* gene have the same phenotype as the one in the cells with the *EFGR* gene overexpressed or kinase cascades activated. 

hTERT overexpression in epithelial cells was shown to increase the content of the fibroblast growth factor (FGF) and the fibroblast growth factor receptor (FGFR) [228, 235]. Moreover, the content of epiregulin, one of the ligands of the epidermal growth factor receptor (EGFR), which plays the key role in maintaining the proliferation status of these cells, is significantly increased in hTERT-immortalized fibroblasts [251]. Epiregulin is known to be repressed in normal human cells; however, it is activated in tumors with high proliferative potential [252]. It is plausible that telomerase stimulates its anti-apoptotic, pro-proliferative, and pro-neoplastic properties. 

Expression of the two isoforms of the vascular endothelial growth factor (VEGF) is activated in human breast cancer cells, as well as in HeLa cells and in the hTERT-transfected normal embryonic lung cells [253]. VEGF is also known to stimulate hTERT expression and activate telomerase via the signal cascades of Ras- and Akt-kinases. Thus, hTERT and the growth factors interact via the positive feedback mechanism in processes of cell cycle regulation, tumor formation, and angiogenesis. 

It was reported in 2003 that telomerase activation can result in the epigenetic silencing of the suppressor genes in cancer cells [254]. The DNA-methyltransferase I promoter ( *DNMT1* ) is activated in normal human fibroblasts upon hTERT expression. The mechanism underlying this effect has not been elucidated yet; however, one can assume that the transcription factor STAT3 is one of the major participant factors in transcription activation. It is already known that STAT3 induces *DNMT1* expression in malignant T-cell tumors [255]. In this case, the signal from hTERT to STAT3 can be transduced by the previously mentioned EGFR, which phosphorylates and thereby activates STAT3 [256]. DNMT1 participates in the regulation of gene expression by methylating the promoter regions of these genes. 

It has recently been established that telomerase also interacts with the Wnt/APC/β-catenin signalling pathway. It has been shown that TERT interacts with the chromatin-remodulating BRG1 factor. BRG1 is the β-catenin co-factor in the processes of the regulation of transcription of the genes associated with the Wnt-signalling pathway. It turned out that TERT can directly interact with the promoters regulated by Wnt and β-catenin. The signalling pathway is known to play a significant role in the cell differentiation and proliferation processes. The effects observed upon TERT expression in stem cells can presumably be explained by the effect of telomerase components on the regulatory cascade [257]. 

## CONCLUSIONS 

Data attesting to the diversity of the functions carried out by the major components of cell telomerase have recently been reported. Some of these functions (such as the nuclease and transferase activities) are associated with the major role of telomerase and its polymerase activity. The other functions (e.g., regulation of gene expression, protection against apoptosis, and contribution to the DNA response to damage) are not directly associated with polymerase activity. It should be noted that the telomerase content in higher eukaryotic cells is very low; hence, almost all the data have been obtained under conditions of artificial expression of its components. Under such conditions, conclusions can be drawn that are divorced from reality. Researchers from different laboratories obtain inconsistent data, which are difficult to interpret. The inconsistency is most likely a result of the use of different systems and models. Nevertheless, the new data reported allows one to assume that telomerase has a more versatile function, and that its impact on the cell is not confined to the regulation of the length of telomere.  

## Abbreviations

TER: telomerase RNA
hTR: human telomerase RNA
TERT: telomerase reverse transcriptase
